# The feature and significance of lower limb MRI in adult myositis patients with anti-NXP2 antibody: a retrospective cohort study in China

**DOI:** 10.3389/fmed.2025.1581902

**Published:** 2025-08-25

**Authors:** Li Shanshan, Wang Lu, Sun Chao, Zhang Ling, Duan Jianghui, Zhu Jianyu

**Affiliations:** ^1^Department of Rheumatology, Key Laboratory of Myositis, China-Japan Friendship Hospital, Beijing, China; ^2^Department of Neurology, China-Japan Friendship Hospital, Beijing, China; ^3^Department of Radiology, China-Japan Friendship Hospital, Beijing, China; ^4^School of Sport and Exercise Rehabilitation, Jinzhou Medical University, Liaoning, China

**Keywords:** anti-nuclear matrix protein 2 antibody, myositis, magnetic resonance imaging, lower limb, clinical feature

## Abstract

**Objective:**

This study aimed to describe the MRI features of lower limbs (thighs and calves) in patients with anti-NXP2 antibody positive myositis, and explore their relationship with clinical manifestations and prognosis.

**Methods:**

Adult patients with anti-NXP2 antibody who underwent both thigh and calf MRI examinations simultaneously were enrolled between 2017 and 2023. The MRI features and medical records of patients were reviewed. Statistical analysis was conducted by SPSS 21.0.

**Results:**

A total of 48 patients (29 females and 19 males) were included in the study. There were fifteen and seven patients with subcutaneous edema on the thigh and calf MRI, respectively. The incidence of fascia edema in the thigh was higher than that in the calf (50.0% vs. 22.9%, *p* = 0.006). All patients experienced varying degrees of thigh muscle edema, with 41 cases (85.4%) showing calf muscle edema on MRI. The primarily affected group in the thigh was the anterior muscle, while the anterior and posterior groups of the calf were equally affected. In addition, the inflammation score of muscle MRI was positively correlated with disease activity (*r* = 0.316, *p* = 0.029), the level of muscle enzymes, serum ferritin (*r* = 0.439, *p* = 0.002), D-dimer (*r* = 0.410, *p* = 0.004), and NSE (*r* = 0.420, *p* = 0.006). Patients with diffuse pattern on MRI generally exhibited higher disease activity. However, there was no difference in the frequency of relapse and mortality between patients with and without diffuse lesions on MRI.

**Conclusion:**

Lower limb MRI of patients with anti-NXP2 antibody provided useful information in evaluating the extent and distribution of lesions. In addition, the degree of muscle edema on MRI was significantly correlated with various clinical features.

## Background

Dermatomyositis (DM) with anti-nuclear matrix protein 2 (anti-NXP2) antibody is a rare autoimmune disease characterized by severe muscle weakness and subcutaneous calcification (1–3). The muscle injury is unique and distinct from other subtypes of DM, such as extreme muscle weakness, significant involvement of distal limbs, and severe microvascular lesions in muscle pathology (4, 5).

For evaluating muscle injuries, manual muscle testing (MMT) has poor objectivity, while muscle biopsy is invasive and may not universal in some cases. In contrast, skeletal muscle magnetic resonance imaging (MRI), as a more objective and non-invasive method, can assist clinicians in evaluating patients’ condition. Previous studies indicated that muscle MRI in DM patients had specific characteristics and diagnostic value (6). In addition, the pattern of muscle MRI may be related to prognosis (7).

As is well known, there is heterogeneity in muscle injury among patients with anti-NXP2 antibody (8). The degree of muscle damage ranges from severe muscle weakness to normal muscle strength. Moreover, the pathological features of muscle biopsy are different, although they share the same specific myositis antibody (MSA). This clinical study aimed to explore the MRI features of patients with anti-NXP2 antibody and their relationship with clinical manifestations and prognosis, which will help clinicians fully understand the value of MRI examination in this particular subtype DM.

## Methods

### Patients

We reviewed the medical records of patients with anti-NXP2 antibody, who were admitted to the Department of Rheumatology at China-Japan Friendship Hospital from 2017 to 2023. In our cohort, the diagnosis of DM met the criteria proposed by European Neuromuscular Centre (ENMC) in 2018, and possible DM sine dermatitis (DMSD) met the 2004 ENMC criteria (9, 10). Patients with onset age greater than or equal to 18 years old were included in the study. In addition, MRI of both thigh and calf should be performed simultaneously. The study protocol was approved by the Ethics Committee of China-Japan Friendship Hospital (approval number 2016–117).

### Clinical data

Patients’ demographic and clinical features were gathered through a systemic record review. Their laboratory data within one week before or after the MRI examination were recorded.

Clinical manifestations included muscle weakness, cutaneous involvement, dysphagia, arthritis, and interstitial lung disease (ILD). Severe muscle weakness was defined as difficulty moving against gravity within 1 month from the onset of disease, according to the Medical Research Council 5-point scale. Muscle strength was measured using the MMT8 proposed by the International Myositis Outcome Assessment Collaborative Study. Cutaneous features included the DM specific rashes (heliotrope rash, Gottron sign, and Gottron papules). Dysphagia was recorded based on patient’s descriptions. Arthritis was recorded based on patient’s descriptions of pain and swollen joint. ILD was diagnosed based on the high-resolution computed tomography (HRCT) image.

Laboratory data consisted of a routine blood test, lymphocyte subsets, the muscle enzyme profile (alanine-aminotransferase - ALT, aspartate-aminotransferase - AST, creatine kinase - CK, lactate dehydrogenase - LDH), albumin, pre-albumin, complement (C3 and C4), C-reactive protein, serum ferritin, tumor markers (carbohydrate antigen 724 - CA724, carcinoembryonic antigen - CEA, cancer antigen 125 - CA125, carbohydrate antigen 199 - CA199, carbohydrate antigen 153 - CA153, neuron-specific enolase - NSE, cytokeratin 19 fragment antigen 21 - CYFRA211, pro-gastrin-releasing peptide - ProGRP, and squamous cell carcinoma antige - SCC), and antinuclear antibody.

Disease activity was evaluated based on the overall assessment by clinicians. High score on visual analog scale (VAS, score range 0–10 cm) indicated severe disease activity. The final assessment of disease activity was estimated based on the mean scores of two rheumatologists.

### Assessment of MRI

MRI examination of the thigh and calf used a 3 T MRI scanner (GE Discovery MR 750, United States). All patients underwent MRI scanning using: axial T1 - weighted fast/turbo spin echo series (repetition time-TR 500 ms; echo time-TE 10 ms); short-tau inversion recovery - STIR fast spin echo series (TR 6770 ms; TE 70 ms; inversion time 230 ms sequences); and axial T2 -weighted series (TR 4800 ms; TE 85 ms). The analysis of MRI image was performed by a ten-year experienced radiologist and a ten-year experienced neurologist who were blinded to patient history independently.

Edema of the subcutaneous tissue, muscular fasciae, and muscle groups were evaluated using STIR. The fatty infiltration was evaluated on T1 sequences. The degree of muscle edema was determined: absent (score 0); mild, intrafascicular (score 1); mild, intrafascicular, segmented (score 2); mild, intrafascicular, global (score 3), moderate intrafascicular, segmented (score 4); moderate intrafascicular, global (score 5) (11). The mean score of two observers was assigned as the degree of muscle edema.

In this study, we examined the muscle of the thigh and calf. The thigh muscles were divided into the anterior compartment (sartorius, rectus femoris, vastus lateralis, vastus medialis, and vastus intermedius), the medial compartment (gracilis, adductor longus, adductor brevis, and adductor magnus), and the posterior compartment (caput breve and caput longum of the biceps femoris, semimembranosus, and semitendinosus). The calf muscles included the anterior compartment (tibialis anterior, extensor hallucis longus, and extensor digitorum longus), the lateral compartment (peroneus longus and peroneus brevis), and the posterior compartment (caput mediale and caput laterale of the gastrocnemius, soleus, tibialis posterior, flexor hallucis longus, and flexor digitorum longus).

### Antibody detection

The anti-NXP2 antibody assay was performed using the EUROLINE Autoimmune Inflammatory Myopathies Ag (IgG) test kit, according to the manufacturer’s protocol (order no. DL 1530–1,601-4G; EUROIMMUN). The positive control was provided by the test kit and the sample buffer was used as a negative control. EUROBlotOne (EUROIMMUN) was used to detect the signal intensity. The definition of being positive for anti-NXP2 antibodies was a result above the cutoff threshold of 25.

### Statistical analysis

SPSS (version 21.0; IBM) was used for statistical analyses. The Kolmogorov–Smirnov test was used to evaluate the distribution of each continuous parameter. Kappa was used for evaluating the consistency. Statistical differences in each group were calculated with t tests (normal distribution), Mann–Whitney U tests (nonnormal distribution), or chi-square tests. Correlation analysis were conducted by Pearson (continuous data) or Spearman (non continuous data). Data were expressed as mean (SD) or median (IQR). All statistical tests were two-sided, and significance was set at *p* < 0.05.

## Results

### The basic characteristics of patients with anti-NXP2 antibody

A total of forty-eight patients (twenty-nine female and nineteen male) participated in this study, including thirty-eight patients with DM and six patients with DMSD. In addition, four patients had severe muscle weakness and non-specific rash in the course of disease progression, who were clinically diagnosed as DM. They were also included in the study.

The disease onset age of enrolled patients ranged from 18 to 67 years old, with an average age of 43.69 ± 14.95 years old at the time of muscle MRI examination. The duration of disease ranged from 1 month to 612 months, with a median time of 8.5 (4.3, 21.0) months. Among them, six patients did not receive any treatment before the MRI examination. In addition, there were five patients with overlap syndrome, including two, two, and one patient with rheumatoid arthritis, Sjogren’s syndrome, and ulcerative colitis, respectively. The average VAS of disease activity was 4.17 ± 1.74, ranging from 1 to 7. Furthermore, there were two patients who had a history of lung adenocarcinoma in the cohort.

### The MRI features of the thigh and calf

Firstly, we evaluated the consistency of four main MRI indicators (subcutaneous edema, fascia edema, muscle edema, and fatty infiltration) between the radiologist and the neurologist, as shown in Supplementary Table 1. All kappa values were greater than 0.600, indicating acceptable consistency.

### Subcutaneous edema in MRI

On the MRI of thighs and calves, subcutaneous edema was observed in fifteen and seven patients, respectively. The subcutaneous edema on thigh MRI was symmetrical distribution, including each one with only anterior edema and only medial edema, which were also symmetrical. Among the seven patients with subcutaneous edema shown on calf MRI, one patient presented asymmetrical involvement, while the remaining cases showed symmetrical involvement. In the cohort, six patients experienced subcutaneous edema in both the thighs and calves. As for the exact location of subcutaneous edema, it was shown in Supplementary Figure 1. The frequency of subcutaneous edema in the thigh was higher than that in the calf, but the difference was not significant (31.3% vs. 14.6%, *p* = 0.052).

### Fascia edema status in MRI

There were 24 patients with fascia edema on thigh MRI. Among them, 11 patients showed fascia involvement on calf MRI. All affected areas on the MRI were symmetrically distributed. In addition, 19 patients had diffuse involvement in three muscle groups on the thigh MRI. For the specific location of fasciitis lesions, it was shown in Supplementary Figure 2. The incidence of fascia edema in the thigh was significantly higher than that in the calf (50.0% vs. 22.9%, *p* = 0.006).

### Muscle lesion in MRI

**Figure 1:**
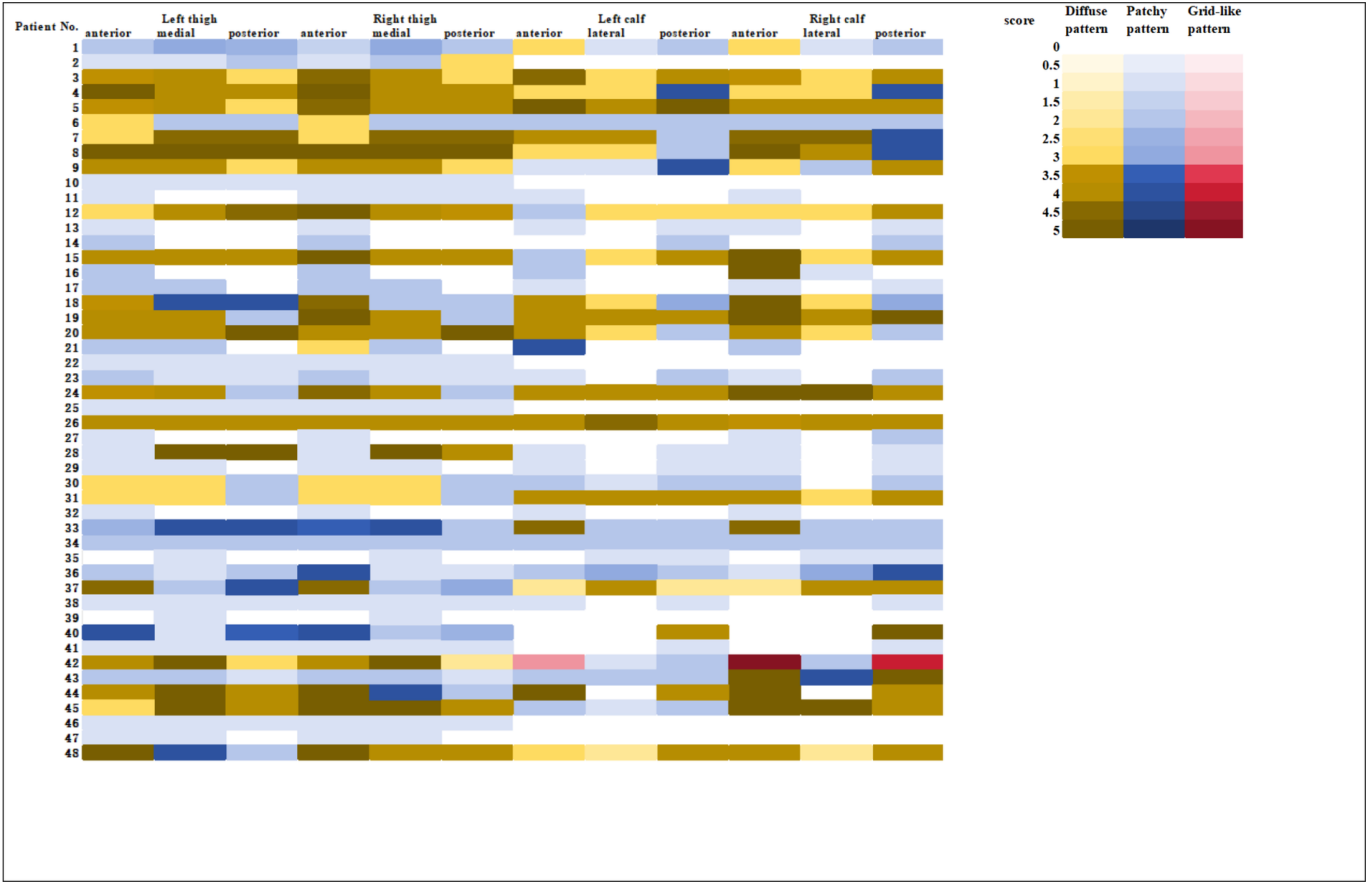
The lower limb muscle inflammation of MRI in patients with anti-NXP2 antibody.

All 48 patients showed varying degrees of muscle edema on MRI, and the detailed data was shown in the heatmap (Figure 1). On the thigh MRI, the anterior muscle group was the most commonly affected with 46 patients. Forty-three and thirty-seven patients had involvement of the medial muscle group and the posterior muscle group, respectively. The primary type of edema was patchy pattern with 24 patients. There were each 12 patients with diffuse or mixed pattern. In terms of the degree of muscle edema, patients with diffuse pattern had the highest scores with 24.58 ± 2.23, followed by mixed pattern with 17.92 ± 4.96 and patchy pattern with 6.92 ± 4.87.

As for the calf muscle, 41 patients (85.4%) showed muscle edema on MRI. Both the anterior and the posterior groups were the primary affected area, with 37 patients in each group. Twenty-seven patients experienced lateral muscle edema. There were 17, 14, and 10 patients with patchy, mixed, and diffuse pattern, respectively. In terms of the degree of calf muscle edema, patients with diffuse pattern had the highest scores with 21.10 ± 5.32, followed by mixed pattern with 17.79 ± 3.91, and patchy pattern with 5.59 ± 3.99.

Considering of the fatty infiltration, 34 out of 48 patients showed an increase in signal intensity in thigh MRI. Contrary to the location of edema in MRI, the primarily affected muscle was the posterior group with 32 patients, followed by the medial group with 29 patients and the anterior group with 17 patients. On the MRI of calf examination, 15 patients had increased signal intensity. The primary involved group was the posterior with 15 patients, followed by the lateral with 11 patients and the anterior with 10 patients.

### Correlation with clinical feature and prognosis

#### The correlation between muscle MRI and clinical manifestations

**Figure 2:**
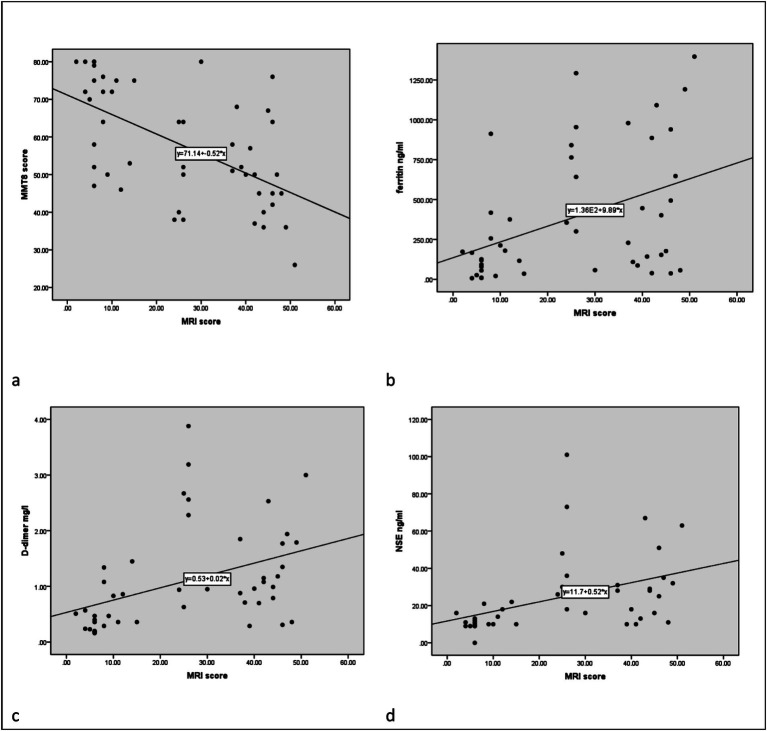
The correlation between MRI inflammatory score and clinical data. The correlation between MMT8 **(a)**, ferritin **(b)**, D-dimer **(c)**, and NSE **(d)** and MRI scores, respectively.

The total score of lower limb MRI was defined as the sum of the thigh and calf muscle inflammation score. We found that younger patients experienced more severe muscle edema. Patients with higher muscle inflammation scores generally exhibited higher disease activity (*r* = 0.316, *p* = 0.029). They had significantly higher levels of muscle enzyme profiles and lower MMT8 score (*r* = −0.577, *p* < 0.001). In addition, they appeared to have more incidence of dysphagia and ILD (both *p* < 0.05). Meanwhile, their PLT levels decreased (*r* = −0.327, *p* = 0.023), while D-dimer (*r* = 0.410, *p* = 0.004) and FDP levels (*r* = 0.446, *p* = 0.002) increased. It was worth noting that the degree of muscle inflammation seemed to be positively correlated with the levels of NSE (*r* = 0.420, *p* = 0.006), which is usually considered as a tumor marker (Table 1 and Figure 2).

**Table 1:** The significant correlation between the inflammatory score of lower limbs muscle MRI and the clinical features.

Clinical feature	*r*	p*
Age enrolled (y)	−0.348	0.015
Proximal limb weakness	0.435	0.002
Distal limb weakness	0.544	<0.001
Severe weakness	0.319	0.027
MMT8	−0.577	<0.001
Dysphagia	0.298	0.039
ILD	0.307	0.034
VAS of disease	0.316	0.029
NK (/ul)	−0.428	0.003
PLT (*10^9^/l)	−0.327	0.023
AST (U/L)	0.467	0.001
CK (U/L)	0.477	0.001
LDH (U/l)	0.370	0.010
Alb (g/l)	−0.400	0.006
C3 (mg/dl)	−0.428	0.002
Fet (ng/ml)	0.439	0.002
D-dimer (mg/l)	0.410	0.004
FDP (ug/ml)	0.446	0.002
CA153 (U/ml)	0.304	0.040
NSE (ng/ml)	0.420	0.006
ProGRP (pg/ml)	−0.376	0.024

*All *p* value less than 0.05.

Next, we divided the patients into two groups according to the presence of diffuse lesion on MRI (Table 2). The frequency of clinical muscle weakness was significantly higher in patients with diffuse lesion, no matter in the proximal or distal limbs (96.6% vs. 63.2, 65.5% vs. 26.3%, *p* = 0.008 and 0.008). Compared with patients with only patchy pattern, patients with diffuse lesions had higher disease activity (3.28 ± 1.71 vs. 4.83 ± 1.42, *p* = 0.002). In terms of laboratory results, patients with diffuse lesions had a decrease of lymphocytes count, especially in the number of NK cells. The levels of muscle enzymes and serum ferritin [424 (118, 926) vs. 120 (27,257) ng/ml, *p* = 0.004] were also significantly higher in patients with diffuse pattern. In addition, patients with diffuse lesion had significantly higher levels of NSE (32.36 ± 23.78 vs. 12.94 ± 6.17 ng/mL, *p* < 0.001), which was not associated with ILD (28.78 ± 23.14 vs. 19.32 ± 16.18 ng/mL, *p* = 0.141) or cancer (42.00 ± 29.70 vs. 23.13 ± 19.58 ng/mL, *p* = 0.072). Although patients with diffuse lesion had more severe conditions, they did not show higher frequencies of relapse and mortality.

**Table 2:** The clinical data between patients with and without diffuse lesion in MRI.

Clinical feature	With diffuse (*n* = 29)	Without diffuse (*n* = 19)	*p*
Gender (F) (*n*, %)	16 (55.2)	13 (68.4)	0.359
Age onset (y)	39.72 ± 13.90	43.21 ± 15.37	0.419
Age enrolled (y)	40.90 ± 13.53	47.95 ± 16.35	0.111
Disease duration (m)	9 (3.5, 19)	8 (6, 32)	0.428
DM (*n*, %)	26 (89.7)	12 (63.2)	0.065
Proximal limb weakness (*n*, %)	28 (96.6)	12 (63.2)	0.008**
Distal limb weakness (*n*, %)	19 (65.5)	5 (26.3)	0.008**
Severe weakness (*n*, %)	22 (75.9)	8 (42.1)	0.018*
Gottrons’ sign (*n*, %)	11 (37.9)	3 (15.8)	0.099
Gottrons’ papules (*n*, %)	9 (31.0)	3 (15.8)	0.394
Heliotrope rash (*n*, %)	22 (75.9)	10 (52.6)	0.095
VAS of rashes	2.55 ± 1.27	1.68 ± 1.20	0.023*
Arthritis (*n*, %)	3 (10.3)	3 (15.8)	0.911
Dysphagia (*n*, %)	24 (82.8)	11 (57.9)	0.058
ILD (*n*, %)	19 (65.5)	7 (36.8)	0.051
VAS of disease	4.83 ± 1.42	3.28 ± 1.71	0.002**
WBC (*10^9^/l)	8.28 ± 3.62	7.37 ± 2.07	0.276
Neu (*10^9^/l)	6.75 ± 3.61	5.61 ± 2.17	0.181
Lym (*10^9^/l)	0.88 ± 0.42	1.19 ± 0.38	0.011*
CD4 (/ul)	443 ± 263	604 ± 334	0.079
CD8 (/ul)	234 ± 175	315 ± 177	0.133
CD4/CD8	2.57 ± 1.43	2.45 ± 1.56	0.797
NK (/ul)	38 (24, 73)	92 (64, 133)	0.001**
B (/ul)	140 (90, 253)	159 (83, 344)	0.575
Hb (g/l)	119.69 ± 17.82	114.37 ± 29.99	0.444
PLT (*10^9^/l)	177.86 ± 62.63	239.47 ± 55.78	0.001**
ALT (U/L)	42 (16.5, 86.5)	24 (18, 42)	0.233
AST (U/L)	68 (27.5, 143.5)	25 (16, 37)	0.001**
CK (U/L)	680 (110, 2337.5)	97 (29, 397)	0.002**
CKmax in history (U/L)	6,367 ± 5,469	3,479 ± 3,981	0.059
LDH (U/l)	511 ± 277	287 ± 68	<0.001**
Alb (g/l)	34.68 ± 3.83	38.03 ± 4.27	0.009**
ProAlb (mg/l)	203.26 ± 63.60	219.01 ± 56.48	0.409
C3 (mg/dl)	77.68 ± 11.07	86.23 ± 17.12	0.064
C4 (mg/dl)	19.37 ± 4.77	18.57 ± 5.31	0.590
CRP (mg/dl)	0.58 (0.19, 0.95)	0.23 (0.16, 0.94)	0.337
Fet(ng/ml)	424 (118, 926)	120 (27, 257)	0.004**
Fib (g/l)	3.26 ± 0.88	3.37 ± 1.22	0.729
D-dimer (mg/l)	1.40 ± 0.95	0.63 ± 0.61	0.002**
FDP (ug/ml)	3.70 (2.13, 6.64)	2 (2, 3.38)	0.003**
CA724 (U/ml)	2.18 (1.24, 7.37)	3.77 (2.18, 9.49)	0.194
CEA (ng/ml)	1.10 (0.80, 2.03)	2.00 (0.79, 2.96)	0.212
CA125 (U/ml)	8.81 (6.67, 13.50)	9.25 (5.17, 13.67)	0.893
CA199 (U/ml)	10.74 ± 9.16	13.64 ± 7.22	0.239
CA153 (U/ml)	13.12 ± 6.09	10.31 ± 3.71	0.087
NSE (ng/ml)	32.36 ± 23.78	12.94 ± 6.17	<0.001**
CYFRA211 (ng/ml)	3.09 ± 1.77	2.47 ± 1.25	0.230
ProGRP (pg/ml)	27.69 ± 11.67	35.75 ± 11.32	0.052
SCC (ng/ml)	0.94 (0.63, 1.48)	0.60 (0.31, 1.20)	0.199
ANA positivity (*n*, %)	14 (48.3)	9 (47.4)	0.951
MRI score	36.76 ± 11.49	8.68 ± 6.33	<0.001**
GC pulse (*n*, %)	7 (24.1)	3 (15.8)	0.739
Relapse^a^ (*n*, %)	19 (70.37)	13 (72.22)	0.893
Relapse in 1st year (*n*, %)	11 (42.3)	5 (27.8)	0.325
Cancer (*n*, %)	2 (6.7)	0	0.512
Death^b^ (*n*, %)	4 (16.7)	0	0.216

*p < 0.05; **p < 0.01.

^a^*n* = 45.

^b^*n* = 41.

#### The comparison between patients with and without calf muscle inflammation

Although the typical manifestation of DM is a decrease of muscle strength in proximal limb, a large number of patients with anti-NXP2 positive myositis had calf muscle involvement, at least on muscle MRI in our cohort. We divided the enrolled patients into two subgroups according to the presence of calf muscle inflammation or not Supplementary Table 2. Patients with calf edema on MRI had higher levels of muscle enzyme profiles, including AST, CK, and LDH. They also showed increased levels of D-dimer (1.20 ± 0.94 vs. 0.47 ± 0.21 mg/L, *p* < 0.001) and NSE (27.11 ± 21.66 vs. 11.43 ± 2.51 ng/mL, p < 0.001). Patients with calf inflammation had lower levels of albumin when compared with those without (35.40 ± 4.16 vs. 39.40 ± 3.57 g/L, *p* = 0.031).

## Discussion

This is a retrospective study on the features of both thighs and calves MRI in patients with anti-NXP2 antibody positive myositis. The anterior muscle group was the primary affected area on MRI. Patients with diffuse lesions on MRI usually had more severe conditions. It was worth noting that we have found a correlation between MRI inflammatory status and levels of NSE for the first time.

In our cohort, the MRI of lower limb muscles in patients with anti-NXP2 antibody myositis generally showed bilateral symmetrical involvement on the locations and patterns, which is consistent with previous studies (12). However, it should be noted that the degree of the lesions may be not strictly symmetrical. In this study, over one-third of patients had asymmetrical inflammation degrees of lower limb lesions (MRI edema score). The anterior muscle group was the primarily affected in the thighs in our cohort, while the medial and posterior thigh muscles seemed more likely to be involved in patients with anti-MDA5 antibody (7). Although there have been many studies on thigh MRI in patients with DM, the research on calf muscle MRI are relatively few. We found that a high proportion of patients had calf involvement, with nearly 85% of patients showing edema on calf muscle MRI. Although some patients did not have symptom of calf weakness in clinical practice, inflammation can still be found on calf MRI. Therefore, the involvement of calves in patients with anti-NXP2 antibody may be underestimated. The proportion of muscle involvement in the anterior and posterior groups of the calf was similar, but it was different from that in the thigh. In addition, the incidence of fatty infiltration was highest in the posterior muscle group, and lowest in the anterior muscle group. This was opposite to the distribution of inflammatory lesions. Subcutaneous edema is also a typical feature of DM (13). As is well known, patients with anti-NXP2 antibody have a higher proportion of subcutaneous edema (4). However, the percentage of subcutaneous involvement in anti-NXP2 antibody positive myositis was comparable to that of the other MSA positive DM on MRI examination, which was somewhat unexpected (14). In addition, only half of the patients in the cohort had fascia edema, which seemed to be significantly lower than that of patients with positive anti-TIF1γ antibody DM. According to a recently published study, all patients with anti-TIF1γ antibody had myofascial edema, despite the small size of the cohort, which only included 12 patients (15).

Patients with diffuse pattern on muscle MRI usually had higher disease activity. The incidence of muscle weakness and systemic involvement were also higher. A good correlation between muscle MRI edema scores and muscle enzyme spectrum levels was identified, which was consistent with other research findings (16). This indicated that muscle MRI may be an alternative indicator of evaluating disease activity in DM (17, 18). However, this conclusion varies depending on the MSA subtype. In a recently published study, patients with a higher proportion of intramuscular lesions on MRI may have fewer lung lesions, suggesting a more favorable prognosis in anti-MDA5 antibody positive DM (7). Although different inflammation patterns were associated with the severity of muscle injury, they cannot predict the prognosis, which may be related to the active treatment in patients with anti-NXP2 antibody. For more severe patients, clinicians prefer to provide intensive treatment, including steroid pulse and biological agents. This treatment is of great significance in controlling muscle inflammation. In addition, infection and tumors remain the main causes of death in patients with anti-NXP2 antibody positive myositis, thus the severity of muscle MRI alone cannot accurately determine the prognosis (8).

As for laboratory data, we found that MRI muscle edema scores were correlated with some inflammatory markers and coagulation related indicators. Patients with high muscle edema scores usually had elevated levels of serum ferritin, although the increase was usually slight. Therefore, there may be differences in the value of serum ferritin between patients with anti-NXP2 antibody and anti-MDA5 antibody (19). The levels of D-dimer and FDP were positively correlated with muscle edema degree in the study. Previous literature also indicated that patients with anti-NXP2 antibody had higher levels of D-dimer (20). This may be related to the unique muscle pathological features. Compared with other MSA positive DM, micro-infarction was a typical pathological feature of anti-NXP2 antibody myositis (5, 21). In addition, the level of NSE was associated with muscle injury in patients, which was an interesting finding. It is currently known that the level of NSE is associated with ILD and cancer in idiopathic inflammatory myopathy (22). Due to some of the enrolled patients with mild ILD in our study, we also conducted a comparative analysis of NSE between patients with and without ILD. The result showed no significant difference. In addition, there was no significant difference in the level of NSE between patients with and without cancer in the study. Therefore, we speculated that the level of NSE may be related to the degree of muscle damage. This may be associated with the inflammatory status and glycolysis processes after muscle injury (23). A previous study has found that elevated serum NSE was observed in patients with active systemic sclerosis, which might be related to platelet activation (24). In our cohort, platelet levels were associated with the inflammatory score of lower limbs muscle MRI, indicating that this possibility cannot be ruled out. However, the exact reason is currently unclear.

It should be noted that this study also has several limitations. Firstly, this was a retrospective study conducted in a single center, including both initial and relapsed patients. In addition, patients with anti-NXP2 antibody also exhibit heterogeneity. Therefore, it may be difficult to completely avoid selection bias. Secondly, we used qualitative and semi- quantitative research methods in the description of MRI, which may not accurately describe the exact condition of MRI. However, the current research findings had practical application value. This method is feasible for radiologists, neurologists, and rheumatologists. Finally, this study only analyzed the muscle MRI features of patients with anti-NXP2 antibody positive, and did not elucidate the associations with pathological characteristics. This will be a further exploration direction for our future research.

## Conclusion

Lower limb MRI of patients with anti-NXP2 antibody provided effective information for evaluating the degree and distribution of subcutaneous, fascia and muscle status. More importantly, the severity muscle inflammation on MRI was closely related to clinical features.

## Data Availability

The raw data supporting the conclusions of this article will be made available by the authors, without undue reservation.
