# Supplementary material for: The feature and significance of lower limb MRI in adult myositis patients with anti-NXP2 antibody: a retrospective cohort study in China
Source: Front Med (Lausanne). 2025 Aug 25;12:1581902. doi: 10.3389/fmed.2025.1581902 (PMC12414990; doi:10.3389/fmed.2025.1581902)
Supplement: Supplementary file 2 [file Image_2.pdf]

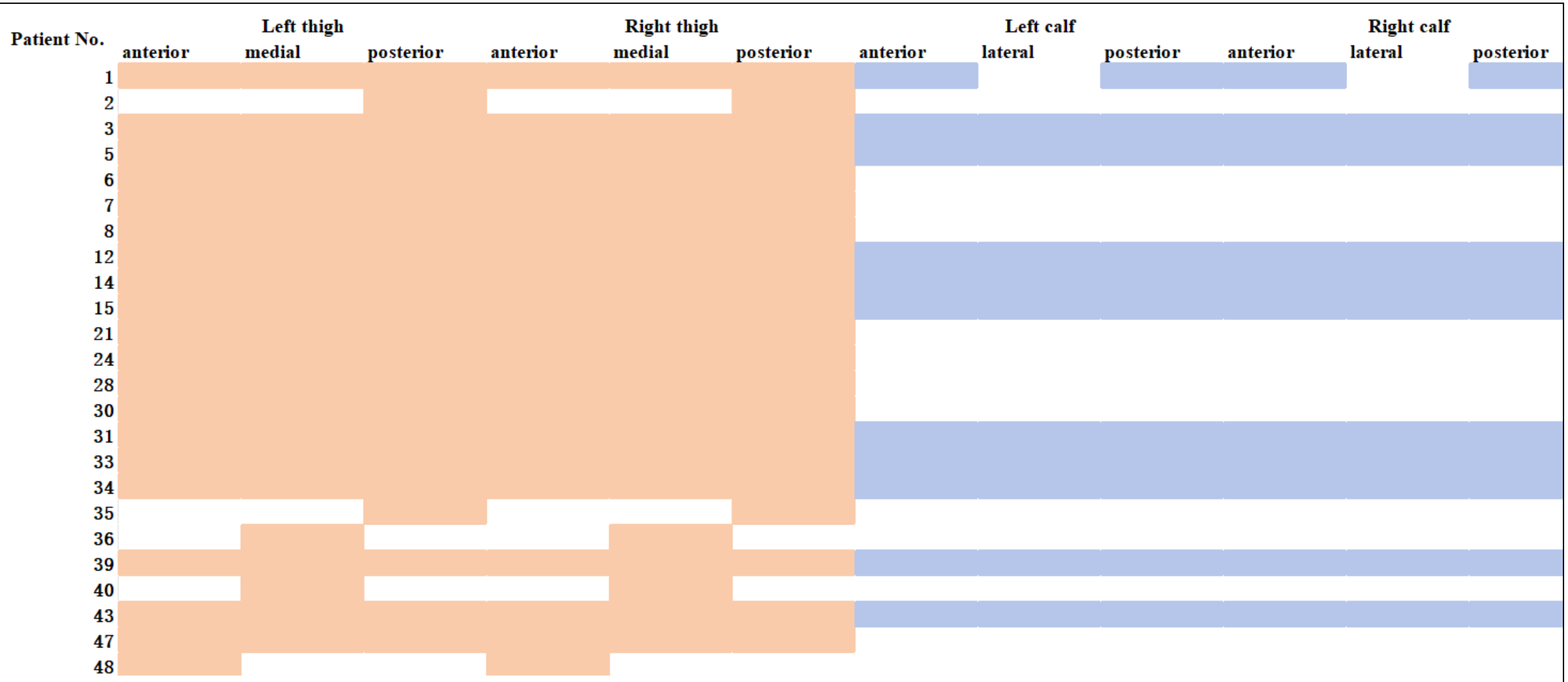

Supplementary Figure 2. the fascia edema of MRI in patients with anti-NXP2 antibody
