# Supplementary material for: The feature and significance of lower limb MRI in adult myositis patients with anti-NXP2 antibody: a retrospective cohort study in China
Source: Front Med (Lausanne). 2025 Aug 25;12:1581902. doi: 10.3389/fmed.2025.1581902 (PMC12414990; doi:10.3389/fmed.2025.1581902)
Supplement: Supplementary file 3 [file Table_1.docx]

Supplementary table 1. the kappa value between the radiologist and the neurologist

|  | Subcutaneous edema | Fascia edema | Muscle edema | Fatty infiltration |
| --- | --- | --- | --- | --- |
| kappa | 0.641 | 0.677 | 0.705 | 0.649 |
