# Supplementary material for: The feature and significance of lower limb MRI in adult myositis patients with anti-NXP2 antibody: a retrospective cohort study in China
Source: Front Med (Lausanne). 2025 Aug 25;12:1581902. doi: 10.3389/fmed.2025.1581902 (PMC12414990; doi:10.3389/fmed.2025.1581902)
Supplement: Supplementary file 4 [file Table_2.docx]

Supplementary table 2. The clinical data between patients with and without calf involvement

|  | With  calf muscle inflammation  (n = 41) | Without  calf muscle inflammation  (n = 7) | P |
| --- | --- | --- | --- |
| Gender (F) | 23 | 6 | 0.219 |
| Age onset (y) | 40.24 ± 14.02 | 46.14 ± 16.98 | 0.323 |
| Age enrolled (y) | 42.05 ± 14.37 | 53.29 ± 15.74 | 0.065 |
| Disease duration (m) | 8 (4, 19) | 21 (6, 50) | 0.091 |
| DM (n) | 31 | 7 | 0.318 |
| Proximal limb weakness (n) | 36 | 4 | 0.080 |
| Distal limb weakness (n) | 24 | 0 | 0.009* |
| Severe weakness (n) | 28 | 2 | 0.086 |
| Gottrons’ sign (n) | 13 | 1 | 0.656 |
| Gottrons’ papules (n) | 10 | 2 | 1 |
| heliotrope rash (n) | 27 | 5 | 1 |
| VAS of rashes | 2.10 ± 1.30 | 2.85 ± 1.21 | 0.156 |
| Arthritis (n) | 4 | 2 | 0.206 |
| Dysphagia (n) | 31 | 4 | 0.370 |
| ILD (n) | 23 | 3 | 0.687 |
| VAS of disease | 4.39 ± 1.67 | 3.17 ± 1.60 | 0.099 |
| Cancer (n) | 2 | 0 | 1.000 |
| WBC (*10^9^/l) | 8.09 ± 3.31 | 6.95 ± 1.04 | 0.088 |
| Neu (*10^9^/l) | 6.49 ± 3.35 | 5.18 ± 1.01 | 0.051 |
| Lym (*10^9^/l) | 0.99 ± 0.44 | 1.07 ± 0.35 | 0.687 |
| CD4 (/ul) | 483 ± 271 | 640 ± 428 | 0.206 |
| CD8 (/ul) | 270 ± 187 | 248 ± 128 | 0.770 |
| CD4/CD8 | 2.46 ± 1.45 | 2.83 ± 1.63 | 0.546 |
| NK (/ul) | 66（28, 99) | 67 (63, 193) | 0.246 |
| B (/ul) | 142 (94, 315) | 116 (60, 344) | 0.377 |
| Hb (g/l) | 118.39 ± 17.05 | 112.86 ± 47.61 | 0.771 |
| PLT (*10^9^/l) | 196.41 ± 62.72 | 236.43 ± 84.15 | 0.145 |
| ALT (U/L) | 40 (17, 73) | 21 (18, 42) | 0.422 |
| AST (U/L) | 53 (24, 118) | 18 (16, 30) | 0.022* |
| CK (U/L) | 397 (106, 1551) | 42 ( 29.80) | 0.001* |
| CKmax in history (U/L) | 5759 ± 5236 | 1852 ± 2133 | 0.080 |
| LDH (U/l) | 452 ± 252 | 250 ± 71 | <0.001* |
| Alb (g/l) | 35.40 ± 4.16 | 39.40 ± 3.57 | 0.031* |
| ProAlb (mg/l) | 207.72 ± 63.85 | 219.62 ± 38.01 | 0.661 |
| C3 (mg/dl) | 78.62 ± 11.99 | 95.34 ± 18.78 | 0.003* |
| C4 (mg/dl) | 19.07 ± 4.64 | 18.95 ± 7.00 | 0.957 |
| CRP (mg/dl) | 0.50 (0.17, 0.90) | 0.22 (0.16, 2.16) | 0.932 |
| Fet (ng/ml) | 278.70 (81.50, 821.75) | 126.00 (10.70, 173.20) | 0.061 |
| Fib (g/l) | 3.27 ± 0.99 | 3.45 ± 1.26 | 0.673 |
| D-dimer (mg/l) | 1.20 ± 0.94 | 0.47 ± 0.21 | <0.001* |
| FDP (ug/ml) | 3.25 (2.02, 5.04) | 2.00 (2.00, 3.41) | 0.070 |
| CA724 (U/ml) | 3.05 (1.44, 8.22) | 3.07 (1.36, 4.12) | 0.939 |
| CEA (ng/ml) | 1.15 (0.77, 2.24) | 2.59 (0.95, 2.99) | 0.215 |
| CA125 (U/ml) | 8.13 (6.12, 13.65) | 10.43 (9.45, 12.1) | 0.490 |
| CA199 (U/ml) | 12.13 ± 8.90 | 10.47 ± 5.96 | 0.640 |
| CA153 (U/ml) | 12.01 ± 5.62 | 12.09 ± 4.52 | 0.972 |
| NSE (ng/ml) | 27.11 ± 21.66 | 11.43 ± 2.51 | <0.001* |
| CYFRA211 (ng/ml) | 3.01 ± 1.67 | 2.08 ± 0.76 | 0.167 |
| ProGRP (pg/ml) | 29.18 ± 11.33 | 37.70 ± 14.08 | 0.115 |
| SCC (ng/ml) | 0.92 (0.50, 1.43) | 0.76 (0.30, 3.88) | 0.664 |
| ANA positivity | 19 | 4 | 0.696 |

* p value less than 0.05
